# Forecasting Influenza Outbreaks in Boroughs and Neighborhoods of New York City

**DOI:** 10.1371/journal.pcbi.1005201

**Published:** 2016-11-17

**Authors:** Wan Yang, Donald R. Olson, Jeffrey Shaman

**Affiliations:** 1 Department of Environmental Health Sciences, Mailman School of Public Health, Columbia University, New York, New York, United States of America; 2 New York City Department of Health and Mental Hygiene, New York, New York, United States of America; Johns Hopkins Bloomberg School of Public Health, UNITED STATES

## Abstract

The ideal spatial scale, or granularity, at which infectious disease incidence should be monitored and forecast has been little explored. By identifying the optimal granularity for a given disease and host population, and matching surveillance and prediction efforts to this scale, response to emergent and recurrent outbreaks can be improved. Here we explore how granularity and representation of spatial structure affect influenza forecast accuracy within New York City. We develop network models at the borough and neighborhood levels, and use them in conjunction with surveillance data and a data assimilation method to forecast influenza activity. These forecasts are compared to an alternate system that predicts influenza for each borough or neighborhood in isolation. At the borough scale, influenza epidemics are highly synchronous despite substantial differences in intensity, and inclusion of network connectivity among boroughs generally improves forecast accuracy. At the neighborhood scale, we observe much greater spatial heterogeneity among influenza outbreaks including substantial differences in local outbreak timing and structure; however, inclusion of the network model structure generally degrades forecast accuracy. One notable exception is that local outbreak onset, particularly when signal is modest, is better predicted with the network model. These findings suggest that observation and forecast at sub-municipal scales within New York City provides richer, more discriminant information on influenza incidence, particularly at the neighborhood scale where greater heterogeneity exists, and that the spatial spread of influenza among localities can be forecast.

## Author Summary

Influenza, or the flu, causes significant morbidity and mortality during both seasonal and pandemic outbreaks. Recently developed influenza forecast systems have the potential to aid public health planning for and mitigation of the burden of this disease. However, current forecasts are often generated at spatial scales (e.g. national level) that are coarser than the scales at which public health measures and interventions are implemented (e.g. community level). Here we build and test influenza forecast systems at county and community levels, which either include spatial connectivity among locations or are run in isolation. We test these four flu forecast systems (i.e. 2 models × 2 spatial scales) using data collected from 2008 to 2013, including the 2009 pandemic, for the five boroughs (corresponding to county level) and 42 neighborhoods (corresponding to community level) in New York City. We compare the performance of the four forecast systems in predicting the onset, duration, and intensity of flu outbreaks and found that the performance varied by spatial scale (borough vs. neighborhood), season (non-pandemic vs. pandemic) and metric (onset, duration, and intensity). In general, the inclusion of spatial network connectivity in the forecast model improves forecast accuracy at the borough scale but degrades accuracy at the neighborhood scale.

## Introduction

Each year, influenza epidemics in the U.S. lead to between 3,000 and 49,000 deaths and cost an estimated $87 billion [1,2]. These significant costs motivate development of forecast systems to predict the epidemic growth of this disease to aid the implementation of more proactive countermeasures. Recent efforts have developed a number of forecast systems for predicting influenza epidemics [3–9]. The skill of each of these systems depends on a number of factors, including: (1) the accuracy and richness of available observational data; (2) the ability of the disease model to capture disease dynamics (should a disease model be part of the forecast system); and (3) the effectiveness of the statistical method used to estimate the data and/or model epidemic features prior to forecast (either statistical characteristics of the epidemic curve or optimized model state-space). Addressing the last factor, our previous work [10] compared the performance of six statistical methods—three ensemble filters and three particle type filters—for the forecast of seasonal influenza epidemics in the US, using the same influenza epidemic model, and revealed key differences among those methods. The model employed for that study depicted influenza transmission dynamics at the municipal scale and had no spatial component. Here we examine whether the inclusion of spatial dynamics improves influenza forecast. In addition, the effect of observation scale, i.e. granularity, on forecast performance is also explored.

Mechanistic models used for disease forecast systems fall into two general categories—compartmental and agent based. Compartmental disease models divide a population into different groups (e.g., susceptible, infected, and removed) according to relevant disease states and apply a set of ordinary differential equations to compute the flow of simulated people among those compartments. In contrast, agent based models simulate the activity and infection state of individuals within a synthetic population based on prescribed schemes of individual behaviors and interactions. While agent based models are more computationally expensive and their model complexity limits their generalizability, compartmental models are often criticized for oversimplifying system dynamics. In particular, compartmental models assume that the entire population (e.g. a country, state, or city) is perfectly mixed; however, even within a city there may be considerable spatial heterogeneity leading to differing disease dynamics at different sub-populations and locations.

Such heterogeneities are evident in S1 Movie, which shows how influenza-like illness (ILI) incidence, as estimated by syndromic surveillance [11], varies in space and time in the 42 neighborhoods of New York City (NYC). Both epidemic intensity and timing differ considerably among the neighborhoods, as disease dynamics are likely driven by both local transmission and inter-locale interaction. Indeed, past studies have shown that commuter flows contribute to the between-state progression of interpandemic influenza epidemics in the US and drive the spatial synchrony among states [12]. As such, the ability of a model to capture spatial connectivity could be an important factor for developing accurate predictive models, as has been demonstrated for a number of infectious diseases [13].

In this study, we investigate how the inclusion of spatial heterogeneity affects the accuracy of influenza forecast for different geospatial scales. Specifically, we address two questions: (1) at what spatial scale (i.e. granularity) of simulation does inclusion of spatial connection improve forecast performance; and (2) whether forecast performance differs between interpandemic and pandemic seasons given the differing transmissibility of influenza strains. To address these questions, we build a forecast system using a patch network epidemic model [14], in conjunction with the ensemble adjustment Kalman filter (EAKF) [15]. The patch network model incorporates spatial connectivity among subpopulations, and each subpopulation is described by a compartmental model. The EAKF data assimilation method is used to optimize the model prior to generating a forecast.

Previous studies have suggested that the product of the ILI consultation rate and laboratory-confirmed influenza positive rate, termed ILI+, provides a more accurate measure of influenza activity and allows more accurate prediction of influenza epidemics [16,17]. Here we test our network forecast system at borough and neighborhood spatial resolutions using ILI+ data collected in NYC from January 2008 to September 2013, including the 2009 pandemic. For each spatial scale, we report the performance of this network model forecast system in comparison to forecasts run in isolation for each locality (referred to as the “isolation” model hereafter).

## Materials and Methods

### Data

Influenza-like illness (ILI) data were collected by the emergency department (ED) syndromic surveillance system of the New York City Department of Health and Mental Hygiene (DOHMH) [11]. This dataset includes weekly ILI syndrome ED visit counts and all-cause ED visit counts at the patient zip-code level, from the week ending 1/12/2008 to the week ending 9/21/2013. ILI syndrome ED visits were defined for patient chief complaints containing a mention of fever and cough, fever and sore throat, or an influenza term [11], analogous to the traditional surveillance definition of ILI as patient presentation with fever ≥37.8°C, a cough or sore throat without a known cause other than influenza [18]. In Staten Island, one of the five NYC boroughs, the majority of ILI syndrome ED visits were recorded only when a visit resulted in hospitalization; as such, ILI syndrome ED visit counts in Staten Island are biased low relative to other boroughs.

As these ILI counts are sparse for each zip-code area, we aggregated the ILI data by borough as well as by United Hospital Fund (UHF) neighborhood, which are similar to community planning district boundaries, and each contain approximately 200,000 people. During the study period, there were 42 UHF neighborhoods in NYC [19]. ILI proportions were calculated as ILI syndrome ED visits divided by total ED visits. Because ILI may include diseases other than influenza, we multiplied the neighborhood or borough ILI proportions by the influenza virus isolation rates for the same week to obtain a more specific measure for influenza (termed ILI+) [16,17]. Influenza virus isolation rates were provided by the World Health Organization and National Respiratory and Enteric Virus Surveillance System (WHO-NREVSS) for Health and Human Service (HHS) Region 2, which includes NYC [20].

The ILI+ time series here include three full inter-pandemic seasons (i.e. from Week 40 to Week 20 of the next year for seasons 2010–11, 2011–12, and 2012–13), two half seasons (data for the 2007–08 season beginning Week 2 of 2008 and for the 2008–09 season truncated at Week 16 of 2009 due to the pandemic), and two pandemic waves (Weeks 14–33 in 2009 for the spring wave and Week 31 in 2009 through Week 20 in 2010 for the fall wave). Note the main epidemic course was captured for both of the two half seasons.

### SIRS-network model

In our previous studies, we used a humidity forced susceptible-infected-removed-susceptible (SIRS) model to simulate the transmission of influenza in US cities [4,10,17,21]. Here we used the same model for simulations in individual boroughs or neighborhoods. The network model includes transmission of influenza within individual locales (either boroughs or neighborhoods) per the humidity forced SIRS model and inter-locale transmission per a gravity type model. Specifically, the network-SIRS model is described by the following equations:

$$\frac{dS_{i}}{dt}=\frac{N_{i}-S_{i}-I_{i}}{L}-S_{i}\sum_{j}^{n}\frac{\beta_{j}(t)c_{ji}{\hat{I}}_{j}}{{\hat{N}}_{j}}-\alpha$$ (1)

$$\frac{dI_{i}}{dt}=S_{i}\sum_{j}^{n}\frac{\beta_{j}(t)c_{ji}{\hat{I}}_{j}}{{\hat{N}}_{j}}+\alpha -\frac{I_{i}}{D}$$ (2)

where *S*_*i*_, *I*_*i*_ and *N*_*i*_ are, respectively, the numbers of susceptible, infected, and total residents in locale *i*; *n* is the total number of locales (5 for the borough-level model and 42 for the neighborhood-level model in this study); *α* is transmission from outside the network domain and is nominally set to 1 per 10 days for all locales; *D* is the infectious period and *L* is the immune period; *c*_*ji*_ is the proportion of residents of locale *i* visiting locale *j* (given in detail below); ${\hat{N}}_{i}$ is the number of people *present* in locale *i* (i.e. including local residents and visitors) and is calculated as ${\hat{N}}_{i}=\sum_{j=1}^{j=n}c_{ij}N_{j}$. Similarly, ${\hat{I}}_{i}$ is the number of infected people *present* in locale *i*. For influenza, viral shedding can start before symptom onset and even after symptom onset, people may continue to travel (e.g. to work); in addition, asymptomatic infection is common [22–24]. Consequently, we assumed that those infected have the same mobility as others and calculated ${\hat{I}}_{i}$ as ${\hat{I}}_{i}=\sum_{j=1}^{j=n}c_{ij}I_{j}$.

The basic reproductive number at time *t*, *R*_*0*_(*t*), is computed using a humidity forcing function [4]:

$$R_{0}(t)=e^{-180q(t)}(R_{0\max}-R_{0\min})+R_{0\min}$$ (3)

with *q*(*t*) as the specific humidity at time *t*, and *R*_*0max*_ and *R*_*0min*_ as the maximum and minimum daily basic reproductive number. The same *R*_*0*_(*t*) values were used for all locales of NYC, as the spatial variability of daily humidity is not great across the city, particularly relative to synoptic and seasonal fluctuations. Further, as daily specific humidity is not available for real-time forecast, we instead used daily climatological specific humidity averaged over 1979–2002 [17,25], which captures the average seasonal cycle, both during model optimization and forecast.

*R*_*0*_ is linked to the transmission rate, *β*, through the expression *β*(*t*) = *R*_*0*_(*t*)/*D*. To account for potential differences in transmission rates among locales (e.g. due to differences in mixing patterns), we multiplied the citywide value by an adjustment factor, *f*_*i*_ for the *i*th locale, to compute the locale-specific transmission rate *β*_*i*_ = *f*_*i*_ *β*(*t*).

In NYC, working commuters comprise the main flow of population among boroughs, and this flow changes over time [26]. Accordingly, we formulated the connections between locations based on commuter flow patterns. Commuter flows are much lower at night on weekdays as well as during the weekend [26]; therefore, for these low-flow time periods, we set *c*_*ij*_ to 0 (for all *i*≠*j*) and *c*_*ii*_ to 1, which reduces the network model to a simple SIRS construct for each locale. During weekday daytime when mass work commute is in effect, we computed *c*_*ij*_ using the following formula:

$$c_{ij}=\frac{p_{i}^{\tau_{1}}p_{j}^{\tau_{2}}/d_{ij}^{\rho }}{\sum_{i=1}^{i=n}p_{i}^{\tau_{1}}p_{j}^{\tau_{2}}/d_{ij}^{\rho }}$$ (4)

where *p*_*i*_ is the population density in locale *i*; *d*_*ij*_ is the distance between locales *i* and *j* (*i*≠*j*); for *i* = *j*, i.e. within locale mobility, *d*_*ii*_ is calculated as $\sqrt{(Area\;of\;locale\;i)/\pi }$, a proxy of the radius for locale *i*; the exponents *τ*_*1*_, *τ*_*2*_, and *ρ* together determine the connectivity between any two locales [12,27].

In the network model, the distances (*d*_*ij*_’s), population sizes and densities for the NYC boroughs and neighborhoods are computed based on data from [28,29]. The state variables *S*_*i*_ and *I*_*i*_ (*i* = 1 to 5 for borough-level simulation or 1 to 42 for neighborhood-level), the citywide parameters *D*, *L*, *R*_*0max*_, *R*_*0min*_, *τ*_*1*_, *τ*_*2*_, and *ρ*, as well as locale-specific parameters *f*_*i*_ (the adjusting factor used to calculate *β*_*i*_ for each borough or neighborhood) were estimated by the ensemble adjustment Kalman filter (EAKF) as described below.

### Mapping ILI+ measures to model simulated influenza incidence rates

As described in the Data section above, the ILI+ observations were measured as the proportions of ILI+ ED consultations among all-cause ED consultations. It is thus not a measure of per capita incidence rate, as simulated by the model. To compute the model-counterpart of ILI+, $\hat{{ILI}_{+}}$, we divided the model-simulated incidence rate by a scaling factor γ. This scaling factor represents the relative likelihood a person seeks medical attention for any cause to that for influenza infection and achieves the conversion per Bayes’ theorem; the scaling factor was estimated by the EAKF in this study. For detailed derivation of this conversion, please refer to [17,21].

### Network-SIRS-EAKF forecast system

In our influenza forecast system, the dynamic model is optimized prior to the generation of a forecast. This optimization, or ‘training’, process is performed using the EAKF [15], a data assimilation method. In practice, an ensemble of model simulations (*m* = 500 in this study) is initialized at the beginning of an integration (see details below); the model ensemble is then numerically integrated forward per the network-SIRS model equations to compute $\hat{{ILI}_{+}}$, the model estimate of ILI+ for the first week (i.e. the prior); the $\hat{{ILI}_{+}}$ prior is then updated using the first observed ILI+ per the EAKF algorithm. Specifically, the mean of the observed variable ensemble, ${\bar{x}}_{k,post}$, is first updated using the following formula:

$${\bar{x}}_{k,post}=\frac{\sigma_{k,obs}^{2}}{\sigma_{k,obs}^{2}+\sigma_{k,prior}^{2}}{\bar{x}}_{k,prior}+\frac{\sigma_{k,prior}^{2}}{\sigma_{k,obs}^{2}+\sigma_{k,prior}^{2}}z_{k}$$ (5)

where the subscript *k* denotes week, and *obs*, *prior*, and *post*, denote the observation, prior and posterior, respectively; ***z*** is the observed weekly incidence, i.e. ILI+ in this study; ***x*** is the observed variable, i.e., $\hat{{ILI}_{+}}$; ${\bar{x}}_{k,post}$ and ${\bar{x}}_{k,prior}$ are, respectively, the posterior and prior ensemble mean; ***σ***^*2*^ is variance. Each ensemble member, $x_{k,post}^{m}$, is then adjusted towards the ensemble mean as follows:

$$x_{k,post}^{m}={\bar{x}}_{k,post}+\sqrt{\frac{\sigma_{k,obs}^{2}}{\sigma_{k,obs}^{2}+\sigma_{k,prior}^{2}}}(x_{k,prior}^{m}-{\bar{x}}_{k,prior})$$ (6)

Further, all unobserved state variables and parameters are updated according to their co-variability with the observed state variable [30]. After the update, the ensemble is integrated to the end of Week 2 and then is updated again using the Week 2 observation of ILI+. This prediction-update filtering process is repeated up to the last observation prior to the generation of forecast. In so doing, the EAKF recursively optimizes the model system to obtain more truthful estimates of model states and parameters. Following any weekly update, a forecast can be generated by integrating the optimized ensemble of model simulations, i.e. the posterior, to the end of the influenza season.

The model-filter system was initialized by assigning the model variables and parameters with values randomly drawn from the following distributions: *S*_*i*_~Unif [30%*N*_*i*_, 100%*N*_*i*_]; *I*_*i*_~Norm (ILI+ for locale *i* at Week 1, $9\sigma_{1,obs}^{2}$), with negative numbers reset to 0; *D*~Unif [1.5, 7] in days; *L*~Unif [200, 3650] in days; *R*_*0max*_~Unif [1.3, 4]; *R*_*0min*_~Unif [0.8, 1.2]; *f*_*i*_~Unif [0.8, 1.2]; *γ*~Unif [3.3, 10] for locales in Staten Island, where only hospitalized ILI ED visits were recorded and *γ*~Unif [1.7, 3.3] for all other locales; *τ*_*1*_~Unif [1, 2]; *τ*_*2*_~Unif [0.2, 1]; and *ρ*~Unif [2, 8]. The model (either SIRS or SIRS-network) was numerically integrated forward using a 1-day time step; all parameters were specified on a daily time-scale and updated weekly through the EAKF data assimilation process.

The system was run discontinuously for each flu season, i.e., the system was initialized at the beginning of each season and “trained” using observations for the current season up to the week of forecast. For all interpandemic seasons but 2007–08, this initialization occurred at Week 40; the incomplete 2007–08 season was initialized at Week 2; the pandemic waves were initialized when influenza transmission occurred out-of-season. Once the first 4 weeks of observations had been assimilated, forecasts were generated weekly until the end of the season (i.e. Week 20 of the next year for interpandemic seasons or four weeks prior to the last observation for the 2008–09 season and the spring pandemic wave). To account for stochasticity in model initialization, 20 separate ensemble simulations were initialized and used to generate weekly forecasts for each season.

### Baseline forecast

For comparison, we developed an additional set of forecasts using an analog method as baseline. Briefly, the Pearson correlation coefficients between the ILI+ time series for the current season up to latest observation and the same period of each historical season were computed. These correlation coefficients represent the similarity in epidemic timing and intensity between the current season and historical seasons. A prediction of ILI+ for the remainder of the season was then computed as the weighted average of the historical time series that correlated positively with the current season (weights are proportional to the correlation coefficients and summed to 1). For real-time forecast, only available historical record can be used; however, as our study period included only seven outbreaks, here we used records for all seven outbreaks to generate the forecasts, regardless of temporal sequence. Due to the small number of locales at the borough scale (i.e. 5) and outbreaks (i.e. 7), we performed this comparison only at the UHF neighborhood scale (i.e. 42 in total). We developed the weighted average in two ways—one using historical records for the same neighborhood (corresponding to the “isolation” model for the EAKF forecasts) and one using records from all neighborhoods (corresponding to the network model for the EAKF forecasts).

### Forecast evaluation

**Fig 1:**
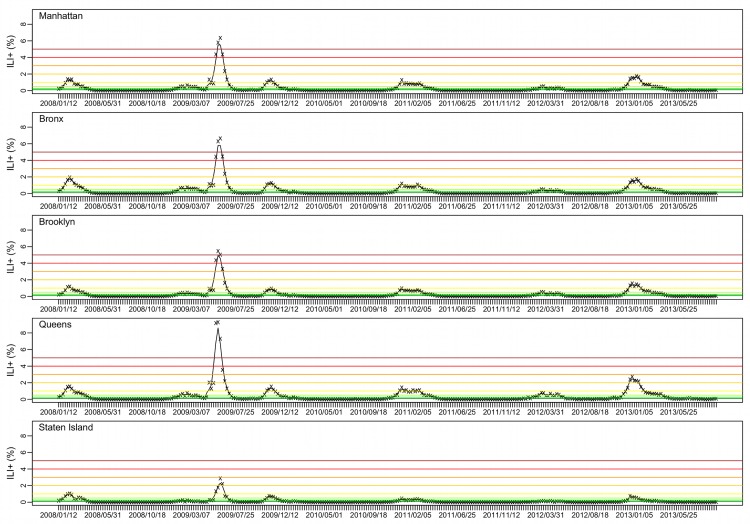
ILI+ for the five NYC boroughs. Weekly ILI+ from the week ending 1/12/2008 to the week ending 9/21/2013 are shown by the ‘x’s; the black trajectory shows the 3-wk centered moving average. Color horizontal lines indicate different ILI+ levels (from the bottom to top: 0.1%, 0.25%, 0.5%, 1%, 2%, 3%, 4%, and 5%).

In our previous studies, we evaluated the accuracy of predictions of epidemic peak timing (i.e. the week with highest ILI+) and peak intensity. In this study, because observations at finer spatial scale may include relatively larger observational errors and thus render the specific week and level of peak incidence less meaningful (e.g. multiple weeks may have similar ILI+ magnitudes, as shown in Fig 1), we here instead evaluated the accuracy predicting the timing and number of weeks at or above various levels of ILI+ (i.e. 0.1%, 0.25%, 0.5%, 1%, 1.5%, 2%, 2.5%, and 3%). These ILI+ levels represent different stages of influenza activity (Fig 1). ILI+ at 0.1–0.25% is close to incidence levels during the onset of an outbreak, 0.5–1% represents incidence at mid-season, 1–2% represents peak levels during inter-pandemic seasons and the Fall 2009 pandemic wave, and higher levels (e.g. 3%) mostly only occurred during the Spring 2009 pandemic wave.

For each level, we evaluated system accuracy predicting the onset (i.e. the first week ILI+ reaches the target level), the duration (i.e. the number of weeks above the target level), and cumulative ILI+ over the duration. To account for observational noise in the data, we computed the 3-week centered moving average for each ILI+ time series and used those moving averages to evaluate the forecasts (referred to as reference hereafter), rather than use the raw ILI+ observations. For onset, a forecast within ±1 week of the reference onset was deemed accurate; for duration, a forecast with a predicted onset within ±1 week of the reference onset and a predicted duration within ±2 weeks of the reference duration was deemed accurate; for cumulative ILI+, a forecast with a predicted onset within ±1 week of the reference onset and a predicted ILI+ sum over the predicted duration within ±20% of the reference value was deemed accurate. Note that the evaluation of duration and cumulative ILI+ takes into account the predicted epidemic timing, in addition to the time span or magnitude; for instance, a forecast with a predicted onset at Week 1 and a predicted duration of 10 weeks will be deemed inaccurate if the observed epidemic started from Week 3 and lasted 10 weeks.

These new forecast metrics will provide users (e.g. public health officials) additional information on the progression of an epidemic that we believe may be more relevant for planning at the local scale. For each metric, we focus our evaluation at shorter leads from 5 weeks (i.e. the event is predicted to occur 5 weeks ahead of the forecast week) to -5 weeks (i.e. the event is predicted to have passed 5 weeks prior to the forecast week). Note forecast accuracy at negative leads is important as well, as it indicates the forecast system is not generating false predictions.

## Results

### Spatial temporal characteristics of influenza activity within New York City

**Fig 2:**
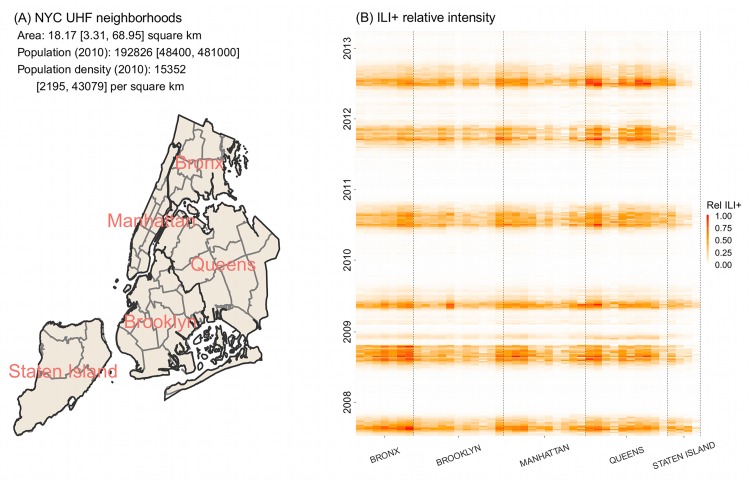
Spatial structure and heterogeneity of ILI+ within New York City. (A) NYC UHF neighborhoods. Borough boundaries are shown in black and UHF neighborhood boundaries are shown in grey. (B) ILI+ intensity relative to the seasonal maximum. Relative ILI+ is computed as the local ILI+ divided by the maximal ILI+ among all neighborhoods for each season/pandemic wave. Each colored square shows relative ILI+ for each UHF neighborhood (x-axis) in each week of the study period (y-axis). Vertical dashed black lines indicate borough divisions.

Fig 1 shows the ILI+ time series over the study period for the five boroughs within NYC. While varied in magnitude, the borough-level ILI+ time series were highly correlated (*r* = 0.955; range: 0.857−0.996), indicating a high level of synchrony among the boroughs. During 2008–2013, NYC was divided into 42 UHF neighborhoods (Fig 2A), each borough with 4 to 11 neighborhoods. At the neighborhood scale, ILI+ varied substantially both in magnitude and timing. As shown in Fig 2B, the epidemic intensity varied dramatically and ILI+ could peak weeks apart (*r* = 0.919; range: 0.347−0.996).

### The performance of forecasts varies by metrics

**Fig 3:**
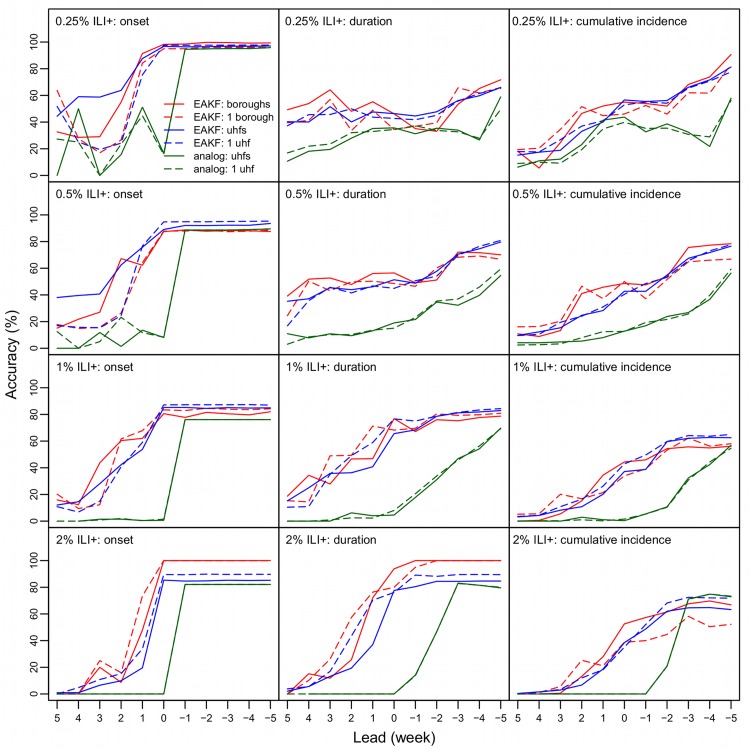
Comparison of forecast accuracy among forecast systems. The six lines in each panel show forecasts made using the borough-level network model and the EAKF (solid red line), the borough-level isolation model and the EAKF (dashed red line), the neighborhood-level network model and the EAKF (solid blue line), the neighborhood-level isolation model and the EAKF (dashed blue line), the baseline analog model with historical records from all localities (solid green line), and the baseline analog model with local historical records (dashed green line). Each row shows the forecast accuracy for different epidemic intensity levels (i.e. 0.25%, 0.5%, 1%, and 2% ILI+) at different leads (x-axis); each column shows the accuracy predicting onset, duration, and cumulative incidence. For onset predictions, the leads are relative to the predicted onset, e.g., 5 weeks in the future (lead = 5), or 5 weeks in the past (lead = −5); for duration or cumulative incidence predictions, the leads are relative to the midpoint of predicted onset and ending. For onset, a predicted value within ±1 week of observed onset was deemed accurate; for duration, a prediction with an onset within ±1 week of observed onset and a duration within ±2 week of observed duration was deemed accurate; for cumulative incidence, a prediction with an onset within ±1 week of observed onset and cumulative incidence over the predicted duration within ±20% of observed cumulative incidence was deemed accurate.

As epidemic intensity varies substantially by season and spatial scale, the same ILI+ level can correspond to different phases of an epidemic. For instance, in the borough of Manhattan, 0.5% ILI+ is roughly the peak of the 2008–09 season but the start of the spring pandemic wave (Fig 1). However, generally, 0.25% ILI+ corresponds to influenza activity at the start of the season, 0.5–1% ILI+ corresponds to elevated influenza activity, and 2% and above ILI+ corresponds to peak levels. Therefore, in general accurate prediction of onset, duration, and cumulative ILI+ at the 0.25% level corresponds to accurate prediction of outbreak onset, duration for the flu season, and cumulative ILI+ over the entire season (1^st^ row in Fig 3); similarly, the accuracies predicting the three metrics at the 0.5% and 1% ILI+ levels (2^nd^ and 3^rd^ rows in Fig 3) and 2% ILI+ level (4^th^ row in Fig 3) reflect the performance of forecast system predicting increased flu activity and peak activity.

Fig 3 shows forecast accuracy for the four forecast systems using the EAKF (network vs. individual locale and borough vs. neighborhood) over the 2007–08 through 2012–13 seasons for four ILI+ levels. All four forecast systems were able to accurately predict onset for different ILI+ levels, and largely outperformed the baseline analog forecast method. Tallied over all weekly ensemble forecasts, forecast accuracies were 81.9% (0.25% ILI+), 73.8% (0.5% ILI+), 55.3% (1% ILI+), and 28.5% (2% ILI+) 1 week prior to the *predicted* onset (i.e. 1 week lead), increased to 97.3%, 91.9%, 86.0%, and 84.9%, respectively, for the four ILI+ levels at 0 lead, and stayed at similar high accuracies at negative leads (i.e. when the onset is predicted to be in the past).

In comparison, forecast accuracy for duration at each level was lower, specifically, with 43.8% (0.25% ILI+), 49.3% (0.5% ILI+), 71.2% (1% ILI+), and 77.1% (2% ILI+) of forecasts accurate at the week the epidemic was predicted to have reached the midpoint of the time-interval ILI+ was above the target level (i.e. 0 lead). Such lower accuracies are not surprising as prediction of duration depends on the ability to predict both the onset and the end point above a given ILI+ level; prediction of the end point is challenging, in particular for low ILI+ levels (e.g., 0.25%) for which the duration could last over 20 weeks (S1 Fig). For the two higher ILI+ levels (i.e. 1% and 2%), for which the outbreak duration tended to be shorter, accuracies were much higher. However, forecast accuracy at all levels improved over time and reached 64.9%, 77.8%, 82.7%, and 84.9% for the four ILI+ levels 5 weeks after the predicted midpoint for the corresponding ILI+ level, which for many localities and seasons was still in advance of the end of the outbreak.

Similarly, forecast accuracy for cumulative ILI+ was lower than for onset. Forecasts of cumulative ILI+ for the duration above a given level had accuracies of 54.18% (0.25% ILI+), 44.8% (0.5% ILI+), 41.3% (1% ILI+), and 40.8% (2% ILI+) at the week the epidemic was predicted to have reached the midpoint of time above target ILI+ level (i.e. 0 lead). These accuracies increased steadily over time and reached 80.1%, 76.2%, 63.0%, 63.9%, respectively, for the four ILI+ levels 5 weeks after the predicted midpoint.

### The impact of spatial connection on forecast performance varies by spatial scale

**Fig 4:**
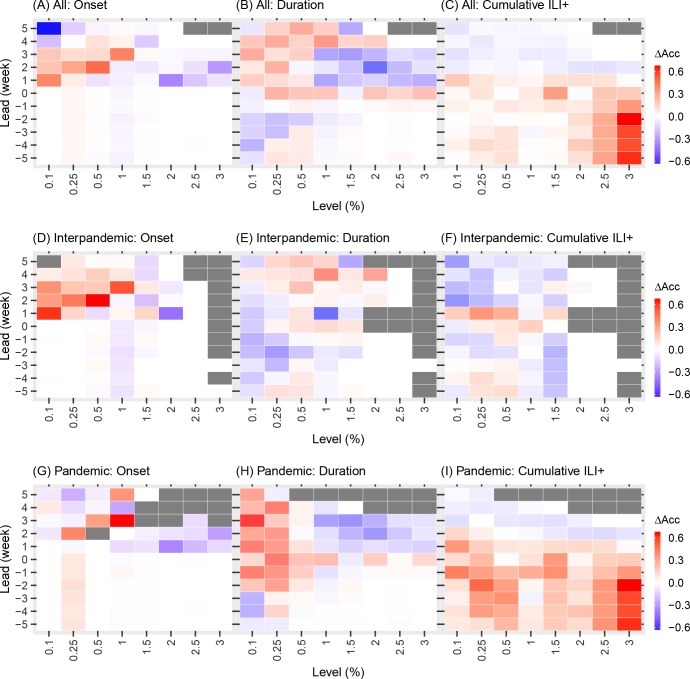
Comparison of the borough level network model and borough level isolation model forecasts. Each colored square shows the difference in accuracy predicting the timing of onset, duration, and cumulative incidence for each ILI+ level (shown on the x-axis) with respect to predicted lead (y-axis). For each bin (i.e. ILI+ level and lead), accuracy difference (ΔAcc), calculated as the accuracy of forecasts using the network model minus those using the isolation model, was averaged over all boroughs and either over all seasons (A-C), inter-pandemic seasons (D-F), or the two pandemic waves (G-I). Grey color indicates insufficient data for bins with <20 forecasts.

**Fig 5:**
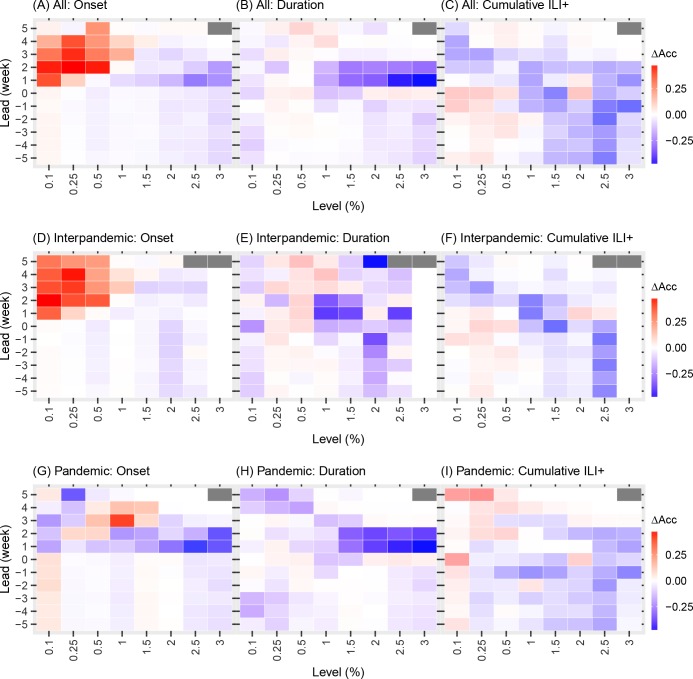
Comparison of the neighborhood level network model and neighborhood level isolation model forecasts. Same notations as in Fig 4.

The network model was better than the isolation model for generating accurate *lead* predictions of onset at lower ILI+ levels (Fig 3 first column and red regions in Fig 4A and 4D and Fig 5A and 5D) at both spatial scales. Inclusion of network structure at the borough level significantly improved forecast accuracy for onset (1.8% higher than those by the forecasts in isolation over all seasons, paired t-test, *P* = 1.92e-7; Table 1) and cumulative ILI+ (1.8% higher, paired t-test, *P* = 0.018) and slightly improved the accuracy for duration (0.9% higher, paired t-test, *P* = 0.116).

**Table 1:** Differences in forecast accuracy using the network model v. the isolation model. Numbers outside the parentheses are the differences in forecast accuracy, in percentage, calculated as the mean accuracy using the network model subtracting that using the isolation model. Numbers in the parentheses are P-values from paired t-tests; shaded cells indicate a significant difference based on a 1-sided paired t-test; pink indicates significantly higher accuracy and blue indicates significantly lower accuracy using the network model. Cells without shading indicate no significant difference based on 2-sided paired t-test. The forecasts were paired by season, lead, and location. The distributions of differences in forecast accuracy are shown in S2 Fig.

		Borough (%)			Neighborhood (%)		
Season	Lead	Onset	Duration	Cumulative ILI+	Onset	Duration	Cumulative ILI+
All	-5:5	1.8 (1.92e-07)	0.90 (0.116)	1.8 (0.018)	4.0 (1.93e-68)	-1.3 (4.35e-06)	-2.4 (3.51e-17)
	0:5	3.2 (2.81e-06)	4.0 (0.000131)	-4.3 (7.69e-05)	8.4 (1.41e-80)	-1.6 (0.000178)	-3.8 (1.39e-19)
	-5:-1	0.61 (0.00995)	-2.4 (0.00856)	8.3 (6.76e-11)	-0.25 (0.0275)	-0.94 (0.00399)	-0.84 (0.0144)
Inter-pandemic	-5:5	2.4 (3.04e-07)	-3.6 (4.9e-06)	-5.4 (1.37e-09)	7.4 (1.83e-123)	-0.68 (0.0257)	-2.9 (9.4e-14)
	0:5	4.6 (2.59e-07)	-1.2 (0.121)	-9.0 (2.97e-12)	14 (5.74e-135)	-1.1 (0.0175)	-6.3 (1.7e-26)
	-5:-1	0 (NA)	-6.2 (4.22e-07)	-1.5 (0.115)	-0.032 (0.414)	-0.21 (0.325)	1.1 (0.0158)
Pandemic	-5:5	0.87 (0.0576)	8.3 (3.45e-09)	14 (2.08e-16)	-2.5 (3.48e-19)	-2.3 (3.41e-06)	-1.6 (3.45e-05)
	0:5	-0.2 (0.418)	13 (1.29e-08)	3.6 (0.0407)	-5.3 (2.83e-19)	-2.5 (0.00152)	0.46 (0.188)
	-5:-1	1.6 (0.00986)	3.6 (0.0143)	24 (7.05e-21)	-0.58 (0.00747)	-2 (9.68e-05)	-3.7 (3.34e-10)

The differences in forecast accuracy at borough scale over different leads are further demonstrated in Fig 4. Tallied over all seasons, prospective forecasts (i.e. positive leads) for onset and duration at lower ILI+ levels (0.1–1%) generated by the borough network model forecast system were more accurate than those generated by the isolation model forecast system (the red regions in Fig 4A and 4B). However, for higher ILI+ levels (1.5–3%), the isolation model forecast system outperformed the network version (blue regions in Fig 4A and 4B). For forecasts of cumulative ILI+ at all levels evaluated, the borough network model forecast system was more accurate at -5 to 1 week leads while the isolation model forecast system was more accurate at 2 to 5 week leads (Fig 4C).

In contrast, the inclusion of network structure at the neighborhood scale in the epidemic model significantly reduced forecast accuracy for most leads and metrics (Fig 5 and Table 1). The main improvement facilitated by inclusion of the network connection among neighborhoods was seen for prospective prediction of onset at the 0.1–1% ILI+ levels (the red region in Fig 5A).

### The impact of spatial connectivity on forecast accuracy differs between interpandemic and pandemic seasons

We additionally examined how the inclusion of network structure in the model affects forecast accuracy during interpandemic seasons and the two pandemic waves. While the inclusion of network structure at the borough scale substantially improved forecast accuracy for duration (8.3%; confidence interval: [5.6%, 11.1%], at -5 to 5 week leads, Table 1 and S2 Fig) and cumulative ILI+ (13.7% [10.5%, 16.9%] at -5 to 5 week leads) during the two pandemic waves, it degraded forecast accuracy for these two metrics during interpandemic seasons (-3.6% [-5.2%, -2.0%] and -5.4% [-7.2%, -3.6%] at -5 to 5 week leads, respectively). In addition, at the borough scale, the network model consistently outperformed the isolation model when predicting onset during both the interpandemic seasons and the two pandemic waves. At the neighborhood scale, the inclusion of network connection significantly improved forecast accuracy for the timing of onset during interpandemic seasons (7.4% [6.8%, 8.0%] at -5 to 5 week leads and 13.9% [12.8%, 14.9%] at 0 to 5 week leads, Table 1 and S2 Fig), despite its generally inferior performance in most other instances (Table 1 and S2J–S2R Fig).

## Discussion

Spatial connectivity has been identified as an important factor shaping the spatial dynamics and progression of infectious disease epidemics [12,13,31]. Here we examine how the inclusion of spatial structure among sub-populations within a municipality influences the performance of an influenza forecast system. We applied a patch network model in conjunction with the ensemble adjustment Kalman filter (EAKF), and weekly ILI+ observations at both the borough and neighborhood levels in NYC and forecast both seasonal and pandemic influenza outbreaks at those two granularities.

The network connection in our forecast system was formulated as a combination of the gravity model [12] and the radiation model [32] (see Methods). In essence, this network connection model computes the raw connections between sub-region pairs (e.g. between boroughs) using a gravity-type formula and further normalizes these raw quantities over the entire region (i.e. all of NYC), which resembles the radiation model. In previous work, we applied this network connection model to sub-regions delineated by population size and area, and used it to compute the spatial connection and infer the transmission network of the 2014–2015 Ebola epidemic in Sierra Leone [14]. Here, we used population density instead of population size as model input. This choice was motivated by the high variability of population density within NYC. For example, population density in Manhattan, the borough that attracts the largest number of commuters [26], is 2.58 times of the city average, even though its residents only make up 19.4% of the city’s population. Using this configuration, our model was able to generate commuter flows similar to those revealed in census data [26] for the majority of boroughs. To test further the impact of potential misrepresentation of the network connectivity, we used commuter census data for the five boroughs in NYC [26] to configure the network connections in the forecast system (see Supplementary Material). The forecasts made using this alternative network configuration had similar accuracy to those made without the commuter census data (paired *t*-test, *P* = 0.2954, Supplementary Material). This finding suggests that our network model is able to capture the impact of spatial connectivity on influenza transmission dynamics in NYC at the borough scale. In addition, the model parameters estimated using the network model were in general consistent with those reported previously for U.S. cities [21] (see Supplementary Material and S3 Fig).

The effects of spatial connectivity on disease transmission are usually two fold—it can introduce a disease into new regions during the early phase of an epidemic [33,34], and it can synchronize disease progression among regions over the course of an epidemic [12,35]. Here we examined both effects in the context of influenza forecast. We found that inclusion of spatial connectivity within a city improved the accuracy of *prospective* forecast of epidemic onset, particularly during interpandemic seasons. This finding underscores the importance of spatial connectivity to disease invasion during the very early phase of an epidemic. In contrast, the effect of inclusion of network structure in the forecast system was mixed in predicting the duration and cumulative ILI+, two later phase metrics. For forecast during the pandemic at the borough scale, the network structure improved prediction accuracy for cumulative ILI+. Influenza moved rapidly through the city during the pandemic, leading to more synchronized dynamics among locales; these synchronized observations probably reinforced the inference and further optimized the system prior to the generation of forecast. However, the network structure degraded the accuracy of cumulative ILI+ for interpandemic events. This finding indicates that the influence of spatial connectivity wanes over the course of a seasonal influenza epidemic and that once the epidemic is established in a locality it is fueled primarily by local transmission dynamics.

The inclusion of network structure and additional data streams in general improved forecast accuracy at the borough level but lowered the accuracy at the neighborhood level. The inferior performance at the neighborhood level may be attributed to two factors: (1) the network model failed to capture the connectivity among neighborhoods, i.e. how local dynamics and heterogeneities propagate across neighborhoods; and (2) assimilation of data streams from outside neighborhoods amplified observational noise, which in turn degraded forecast accuracy. For the former, heterogeneities in epidemic progression at the neighborhood level not only come from differing rates of population mixing (for which the network structure is primarily designed), but also from other factors such as population demographics (e.g. different age-structure and school-age populations) and healthcare seeking behaviors, which affect the number of cases recorded by the surveillance system. These heterogeneities, as captured in the observed ILI records (S1 Movie), likely reflect local dynamics that, in contrast to our network model specification, do not propagate outside the neighborhood. Work to investigate the nature of these neighborhood level heterogeneities is underway.

To test the impact of the latter factor, i.e. confounding from outside signals through data assimilation, we generated forecasts for the 3-wk and 5-wk centered moving averages of the neighborhood ILI+ time series. These smoothed signals are less erratic than the raw observations so that the signal from connected neighboring localities is more stable over time. Indeed, the forecast accuracies for these two smoothed time series were substantially higher than those for the raw observations, and forecasts generated using the network model-EAKF forecast system were more comparable to those run in isolation (S4 Fig).

Together, these findings suggest that the heterogeneities captured in the neighborhood level dataset represent local dynamics or observational biases that do not impinge upon the signal of other neighborhoods. As a result, forecasting at the neighborhood scale is more accurate when each neighborhood is run independently in isolation. In contrast, at the borough scale, these heterogeneities are aggregated and homogenized; our network model, capturing the connectivity via commuter flow, likely reflects the primary inter-connections among boroughs and thus to some extent improved forecast accuracy. For these reasons, our findings here do not entirely dismiss inclusion of spatial heterogeneity among subpopulations in forecast models. Rather, it suggests that to improve the forecast model, the network structure should represent spatial connectivity at the appropriate scale. Indeed, the lack of forecast improvement at the neighborhood scale may simply indicate that alternate, more detailed modeling approaches are needed. Such approaches would need to be supported by richer observation at an appropriate granularity so that critical epidemiological processes, in addition to population mobility, could be represented and optimized.

We recognize a number of limitations in this study. Our conclusions were drawn based on only seven outbreaks; a more robust evaluation is warranted as more observations become available. We did not include age-structure in our model, an important demographic factor shaping influenza transmission dynamics. The ED ILI counts were recorded by age group; however, age-grouped ILI ED counts were too low at the UHF neighborhood level to allow meaningfully analysis. Future work using data from other surveillance systems may resolve this issue. Another potential confounder stemmed from the ED ILI data for Staten Island, one of the five NYC boroughs, which only included hospitalized cases. To address this issue, we used a different prior distribution of scaling factor (*γ*) for localities within Staten Island (see Methods). Our previous work [21] showed that this scaling factor is able to compensate for missing data; it thus may to some extent mitigate adverse impacts on the network model.

Due to a lack of city-specific data, we used influenza viral isolation rates reported for HHS Region 2 to compute the ILI+ for NYC. As viral testing practice may vary by geographical location, the viral isolation rates used here may not be representative of those for NYC and may introduce systematic biases into the ILI+ data. In addition, the ILI+ data used here were based on ED ILI visits and hence may not be representative of influenza activity among the general population. Nevertheless, forecast of ED ILI+ in itself is important as it could provide health professionals at EDs advanced lead-time to plan for changes in influenza-related visits.

In summary, we have developed a network model-filter forecast system for the forecast of influenza at borough and neighborhood geospatial scales. When tested using data for NYC, this network forecast system was able to improve forecast accuracy at the borough level, for which spatial connection is linked primarily via commuter flow. In contrast, it degraded forecast accuracy at the neighborhood scale. NYC is a large municipality with a sizeable geographic extent and many neighborhoods; however, even within smaller cities, community and neighborhood heterogeneity may lead to variable disease transmission dynamics. Local public health measures implemented at sub-municipal scales would therefore benefit from surveillance and forecast enacted at those more local scales. Future work should continue to explore spatial granularity and identify the optimal scales for disease surveillance, forecast and intervention. In so doing, the network model forecast system should be extended to other cities and geographic units (e.g. states) to examine its utility in these different settings and spatial scales.

## Supporting Information

S1 Text Supplementary analyses and results. (DOCX)

S1 Movie Weekly influenza-like illness (ILI) emergency department visit rates for the United Hospital Fund neighborhoods within New York City during of the 2008–2009 influenza season. (MP4)

S1 Fig Distributions of the duration above each ILI+ level and cumulative ILI+ over that duration at the borough (red) and neighborhood (blue) scales. (TIF)

S2 Fig Distributions of differences in forecast accuracy between the network model and the isolation model. Each subplot (A-R) shows the difference in forecast accuracy (in percentage) for forecasts made for the same spatial scale and metric using the network model v. the isolation model, for -5 to 5 week leads (in orange), 0 to 5 week leads (in green) and -5 to -1 week leads (in blue). The forecasts are grouped by season, lead, and location as in Table 1; each subplot corresponds to one cell in Table 1. The numbers (in percentage) in each subplot show the mean and 95% confidence intervals for each of the three lead times.
(TIF)

S3 Fig Parameter estimates made using the four forecast systems: the borough-level network model-EAKF (dark green), the UHF neighborhood-level network model-EAKF (light green), the borough-level isolation model-EAKF (blue), and the UHF-level isolation model-EAKF (light blue). (TIF)

S4 Fig Comparison of forecasts at the neighborhood scale using the raw observations, and 3-wk- and 5-wk- moving average ILI+ time series. Same notations as in Fig 3.
(TIF)
