# Supplementary material for: Forecasting Influenza Outbreaks in Boroughs and Neighborhoods of New York City
Source: PLoS Comput Biol. 2016 Nov 17;12(11):e1005201. doi: 10.1371/journal.pcbi.1005201 (PMC5113861; doi:10.1371/journal.pcbi.1005201)
Supplement: S1 Text — (DOCX) [file pcbi.1005201.s001.docx]

Supplementary Material

Forecasting influenza outbreaks in boroughs and neighborhoods of New York City

Wan Yang^1^, Donald R. Olson^2^, Jeffrey Shaman^1^

^1^Department of Environmental Health Sciences, Mailman School of Public Health, Columbia University, New York, New York, United States of America

^2^New York City Department of Health and Mental Hygiene, New York, New York, United States of America

**1. Testing of network connection formulation**

In our network model, we used a combination of the gravity model [1] and the radiation model [2] to compute the connectivity between locations (e.g. two boroughs or two neighborhoods) during daytime on weekdays when mass work commute is in effect. This formulation provides a relatively realistic representation of the working commuter flow among boroughs, in comparison to census survey data [3]. Table S1 shows the connectivity matrix [*c_ij_*] computed using our model. Using this connectivity configuration, the estimated daytime population in the boroughs within New York City closely matched the census survey data (Table S2). The relative errors were 1.18% for Manhattan, 11.19% for the Bronx, 15.92% for Brooklyn, and 8.30% for Staten Island. However, the relative error was large for Queens (57.32%). This error is largely due to the overestimation of outward commuter flow from Queens to Manhattan (Table S1).

**2. Comparison of forecasts using the network model alone and those using the network model along with commuter census data**

As described above, our network model tends to overestimate the population outflow for the Queens borough. To test the impact of this overestimation, we used the population outflow reported by census survey [3] for each borough and then apportioned each outflow among outside boroughs based on the network configuration (which determined the inflow to each borough from outside boroughs). As shown in Tables S1 (light grey cells) and S2, this method provided more accurate estimates of network connections among boroughs as well as daytime populations for the Bronx, Brooklyn, Queens, and Staten Island.

We then reran the borough level network forecasts using this alternative network configuration. The forecasts made using this alternative network configuration had similar accuracy to those made without the commuter census data (paired *t*-test, *P*= 0.2954). This finding suggests that our network model is able to capture the network structure among subpopulations in NYC, at least at the borough level.

**3. Comparison of parameter estimates by different models**

As described in the Methods in the main text, model parameters (e.g. *D*, the infectious period) were treated as free variables and updated weekly through the data assimilation process. In this section, we compare the estimates for *R_0_*, the basic reproductive number, *R_e_*, the effective reproductive number, *D*, the infectious period, as well as the three parameters in the gravity-like network model (i.e. *τ_1_*, *τ_2_*, and *ρ* from Eqn. 4 in the main text). The first three parameters were used in both the SIRS-network model and the SIRS model for each locality (i.e. “isolation” model), while the latter three were only used in the network model. As the estimates varied by week, we evaluated each parameter estimate at the week when the citywide ILI+ was at its seasonal maximum (i.e. the peak). Of note, in theory, *R_e_* should be close to unity at the epidemic peak; however, some estimates made here were above 1 (S3 Fig), in particular, for seasons 2010-11, 2011-12, 2012-13, and the two pandemic waves. Two factors may have produced this discrepancy: (1) the peak week was defined using aggregate citywide ILI+ rather than local ILI+ and thus might not match the local peak; (2) for some seasons (e.g. 2010-11), ILI+ stayed near peak for several weeks (see Fig 1), which may have affected filter estimation.

As shown in S3 Fig, *R_0_*, *R_e_* and *D* varied by season; and the variations were in general consistent across model constructs and spatial scales. In addition, these estimates were generally consistent with those reported previously for over 100 cities across the US [4]. In comparison, the three network model parameters were more stable across seasons. Not surprisingly, the estimates at the UHF neighborhood scale were more dispersed, as greater variations exist in the UHF level ILI+ data.

References:

1. Viboud C, Bjornstad ON, Smith DL, Simonsen L, Miller MA, et al. (2006) Synchrony, waves, and spatial hierarchies in the spread of influenza. Science 312: 447-451.

2. Simini F, Gonzalez MC, Maritan A, Barabasi AL (2012) A universal model for mobility and migration patterns. Nature 484: 96-100.

3. Moss ML, Qing C (2012) The Dynamic Population of Manhattan. https://wagner.nyu.edu/files/rudincenter/dynamic_pop_manhattan.pdf

4. Yang W, Lipsitch M, Shaman J (2015) Inference of seasonal and pandemic influenza transmission dynamics. Proc Natl Acad Sci U S A 112: 2723-2728.

Table S1. Comparison of network connectivity matrices. Columns in black show results computed using our network model (Eq. 4 in the main text); grey cells show results computed using our network model along with census data [3]. The parameters used are *τ_1_*=1.5, *τ_2_*=0.6, and *ρ*=5; these values are the means of the prior distributions used in this study. Each column shows the distribution of residents from each borough among the five boroughs within New York City during weekday daytime. For example, for Manhattan, using the network model alone, 99.5% of its residents stay in their home borough, 0.4% commute to the Bronx, 0.1% commute to Brooklyn, 0.1% commute to Queens, and nearly none commute to Staten Island (shown as 0 due to rounding).

|  | Manhattan | | Bronx | | Brooklyn | | Queens | | Staten Island | |
| --- | --- | --- | --- | --- | --- | --- | --- | --- | --- | --- |
|  | model | w. data | model | w. data | model | w. data | model | w. data | model | w. data |
| Manhattan | 0.995 | 0.917 | 0.138 | 0.202 | 0.106 | 0.162 | 0.447 | 0.182 | 0.033 | 0.061 |
| Bronx | 0.004 | 0.057 | 0.857 | 0.790 | 0.003 | 0.005 | 0.071 | 0.029 | 0.002 | 0.004 |
| Brooklyn | 0.001 | 0.015 | 0.001 | 0.002 | 0.862 | 0.789 | 0.158 | 0.064 | 0.094 | 0.175 |
| Queens | 0.001 | 0.011 | 0.004 | 0.006 | 0.027 | 0.042 | 0.323 | 0.725 | 0.002 | 0.005 |
| Staten Island | 0.000 | 0.000 | 0.000 | 0.000 | 0.002 | 0.003 | 0.000 | 0.000 | 0.869 | 0.757 |

Table S2. Daytime population sizes in the five NYC boroughs estimated by our network model, in comparison to census survey data. Census data were taken from [3], page 9, Figure “Weekday Commuting Flows by County/Borough, 2009”, and shown in the column “Census data”. The 3^rd^ and 4^th^ columns show the estimates of weekday daytime population in each borough using our network model and the estimation errors relative to the census data; the 5^th^ and 6^th^ columns show the estimates of weekday daytime population in each borough using our network model along with the census data and the estimation errors relative to the census data.

| Borough | Census data | Using the network model | | Using the network model and census data | |
| --- | --- | --- | --- | --- | --- |
|  |  | Estimates | Relative error | Estimates | Relative error |
| Manhattan | 3083873 | 3047395 | 1.18% | 2574572 | 16.51% |
| Bronx | 1223108 | 1360011 | 11.19% | 1262993 | 3.26% |
| Brooklyn | 2207700 | 2559163 | 15.92% | 2227152 | 0.88% |
| Queens | 1865722 | 796220 | 57.32% | 1748546 | 6.28% |
| Staten Island | 380730 | 412343 | 8.30% | 361870 | 4.95% |
