# Supplementary figures and images for: Forecasting Influenza Outbreaks in Boroughs and Neighborhoods of New York City
Source: PLoS Comput Biol. 2016 Nov 17;12(11):e1005201. doi: 10.1371/journal.pcbi.1005201 (PMC5113861; doi:10.1371/journal.pcbi.1005201)

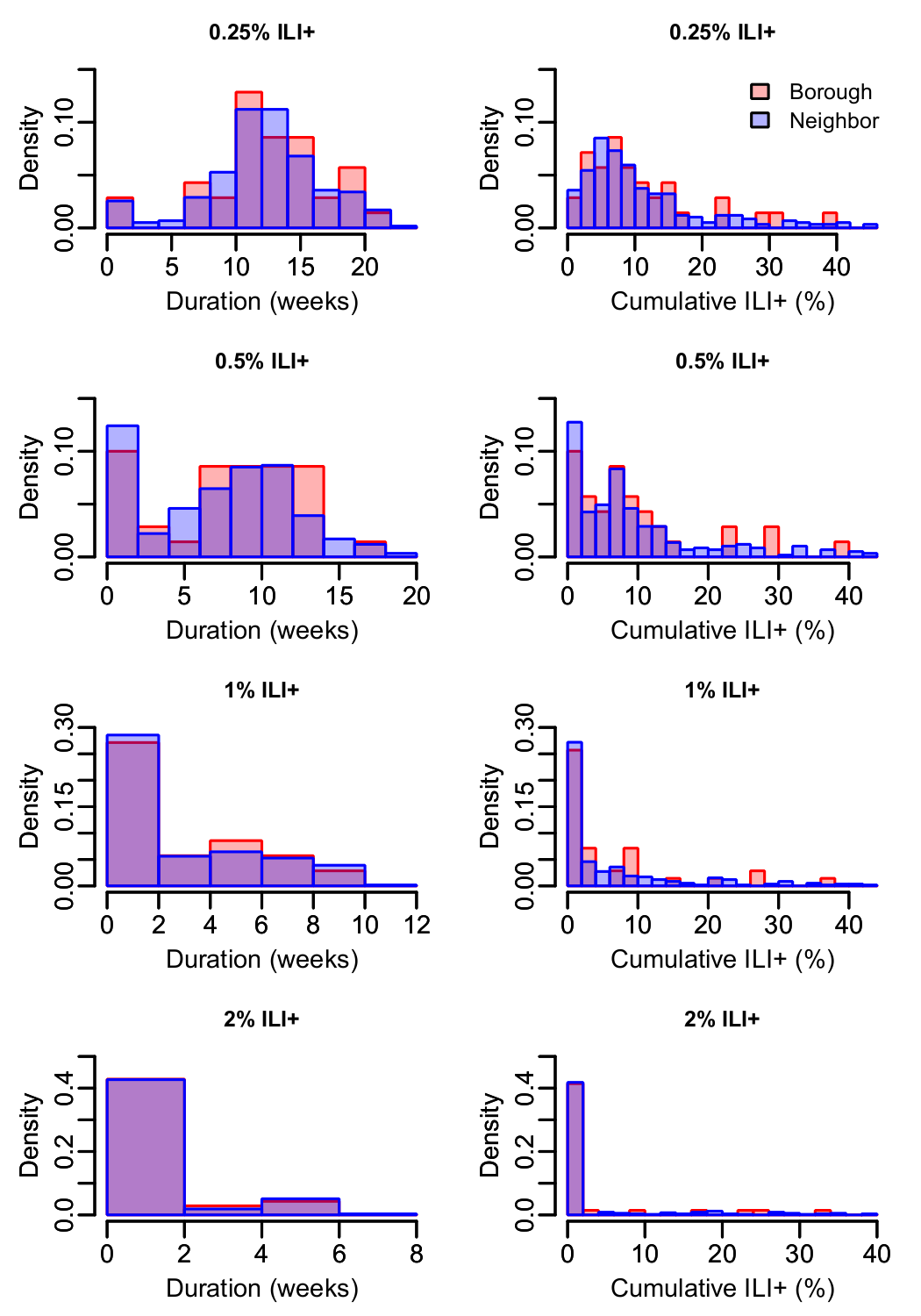

Supplement: S1 Fig — (TIF) [file pcbi.1005201.s003.tif]

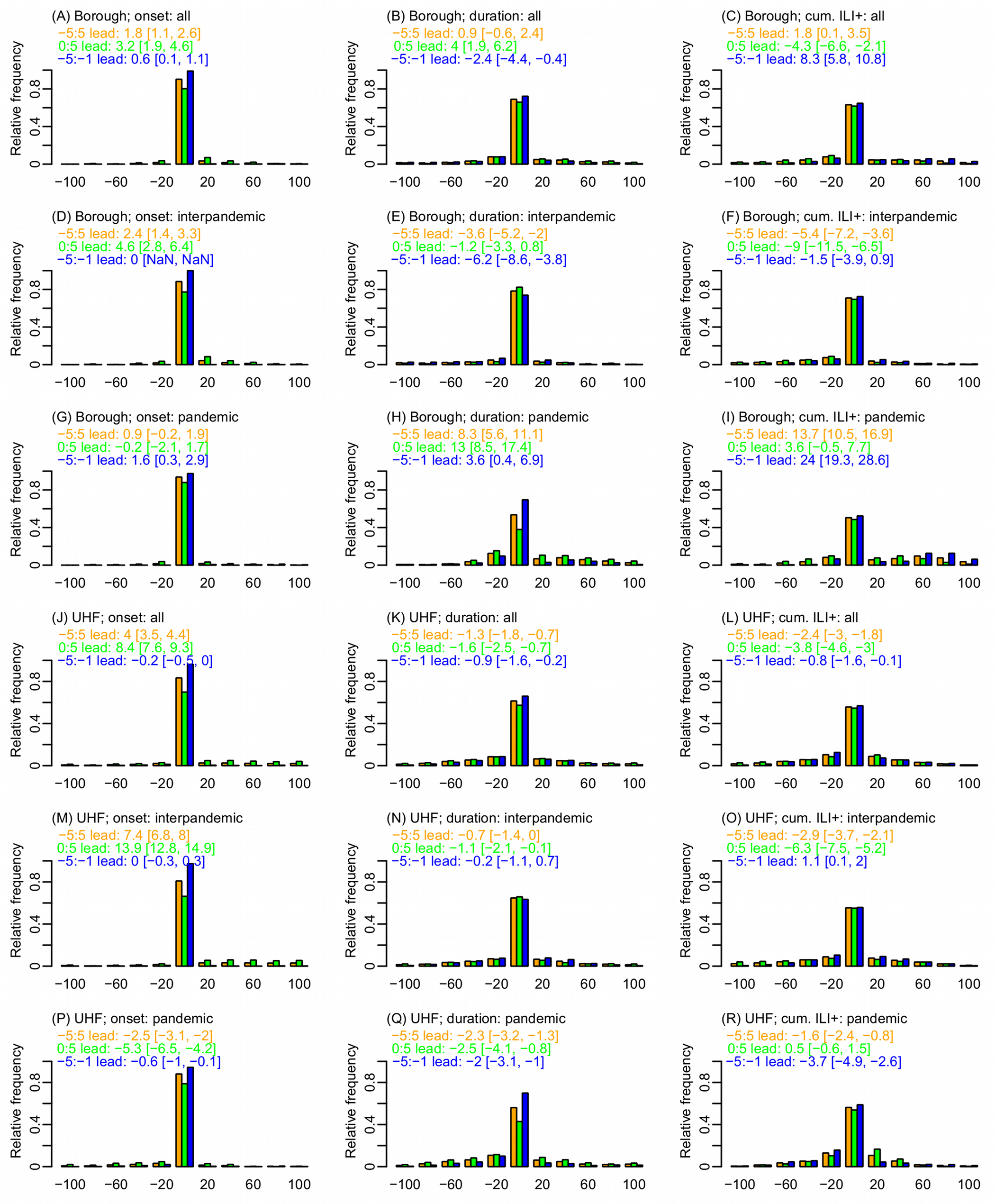

Supplement: S2 Fig — Each subplot (A-R) shows the difference in forecast accuracy (in percentage) for forecasts made for the same spatial scale and metric using the network model v. the isolation model, for -5 to 5 week leads (in orange), 0 to 5 week leads (in green) and -5 to -1 week leads (in blue). The forecasts are grouped by season, lead, and location as in Table 1; each subplot corresponds to one cell in Table 1. The numbers (in percentage) in each subplot show the mean and 95% confidence intervals for each of the three lead times. (TIF) [file pcbi.1005201.s004.tif]

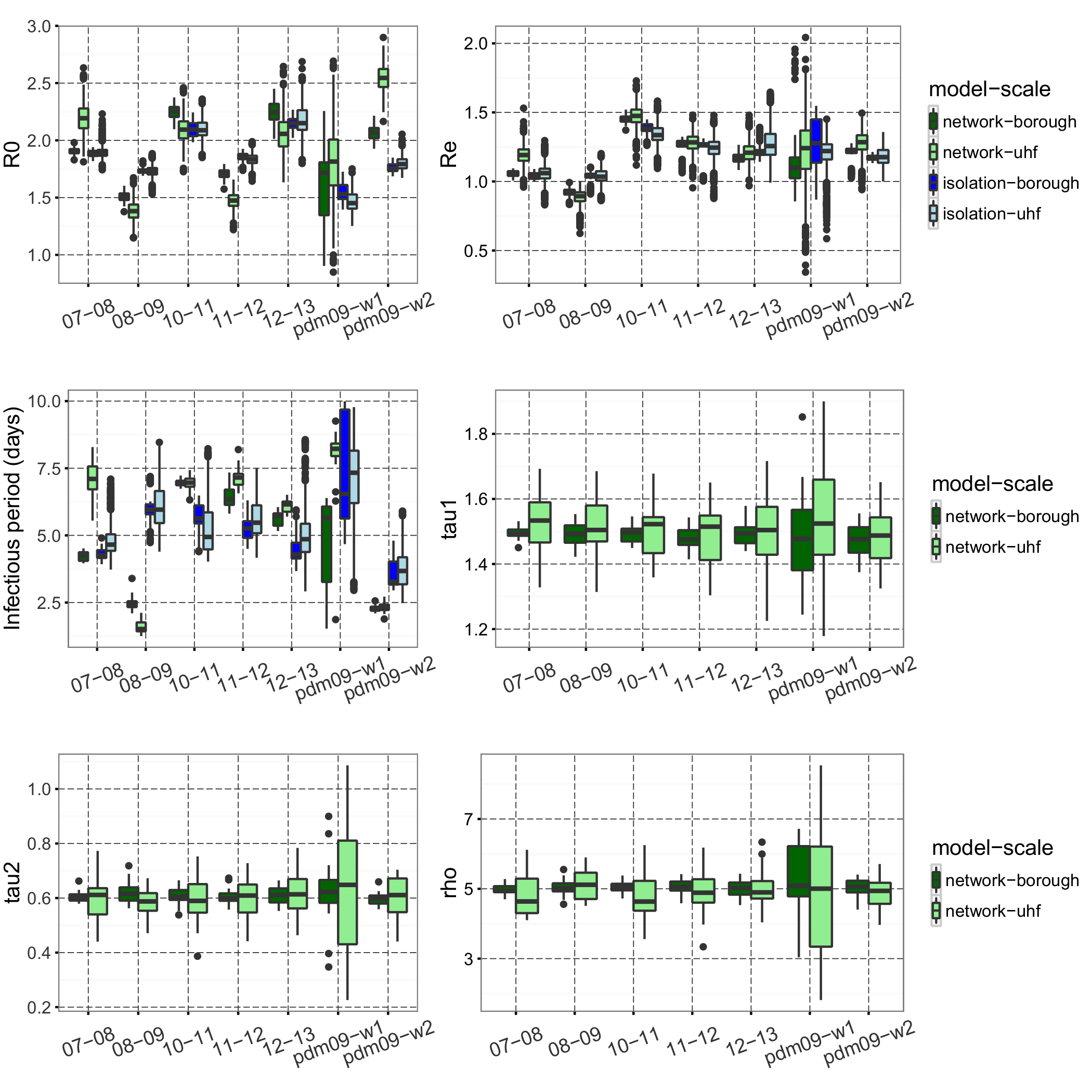

Supplement: S3 Fig — (TIF) [file pcbi.1005201.s005.tif]

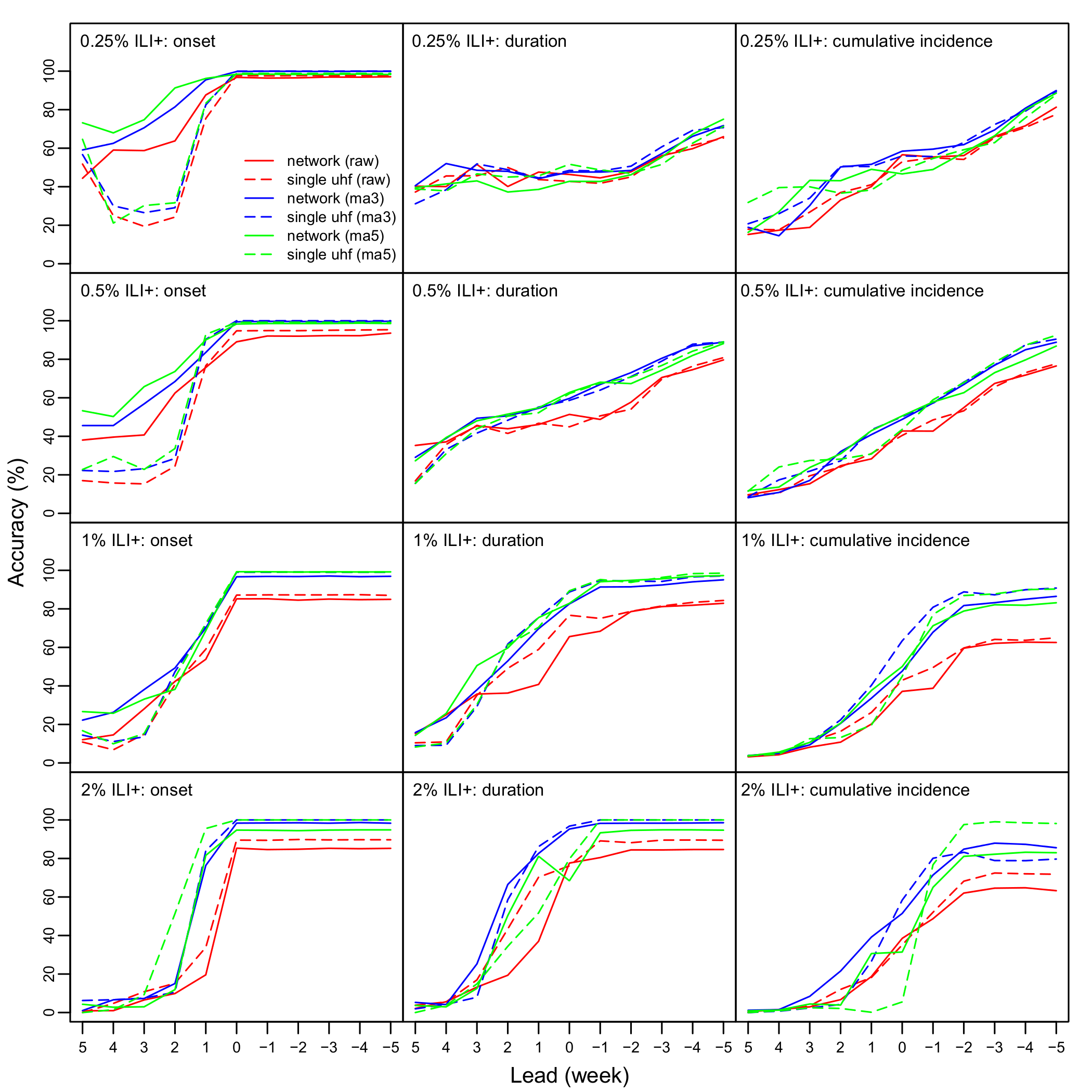

Supplement: S4 Fig — Same notations as in Fig 3. (TIF) [file pcbi.1005201.s006.tif]
